# STAT4 facilitates PD‐L1 level via IL‐12R/JAK2/STAT3 axis and predicts immunotherapy response in breast cancer

**DOI:** 10.1002/mco2.464

**Published:** 2023-12-15

**Authors:** Jianbo Zhou, Feng Wan, Li Wang, Cheng Peng, Ruizhen Huang, Fu Peng

**Affiliations:** ^1^ West China School of Pharmacy Sichuan University Chengdu China; ^2^ State Key Laboratory of Southwestern Chinese Medicine Resources Chengdu University of Traditional Chinese Medicine Chengdu China; ^3^ Department of Cardiovascular Hospital of Chengdu University of Traditional Chinese Medicine Chengdu China; ^4^ Key Laboratory of Drug‐Targeting and Drug Delivery System of the Education Ministry and Sichuan Province Sichuan Engineering Laboratory for Plant‐Sourced Drug and Sichuan Research Center for Drug Precision Industrial Technology Sichuan University Chengdu China

**Keywords:** breast cancer, immunotherapy, PD‐L1, STAT4

## Abstract

Signal transducer and activator of transcription 4 (STAT4) is a critical transcription factor for T helper cell differentiation and tumor cells. Although its prognostic role and gene function have been reported in several carcinomas, the role of STAT4 in vitro and in vivo in breast cancer remains poorly understood. The effect of STAT4 in immunotherapy is also unclear. Therefore, we integrated bulk transcriptomics, experiments, and single‐cell transcriptomics to systematically analyze its function in prognosis and signaling pathway. Several clinical breast cancer cohorts confirmed STAT4 as a T‐cell relevant prognostic biomarker. Overexpressed STAT4 increased programmed cell death ligand 1 (PD‐L1) and major histocompatibility complex class II levels in breast cancer cells. In molecular mechanism, transcriptional synergy between STAT4 and STAT3 transactivated interleukin (IL)‐12R and involved a positive feedback loop: STAT4/IL‐12R/JAK2–STAT3–STAT4, which contributed to the upregulation of PD‐L1 expression. The above signaling axis was defined as the STAT4‐related pathway and its score was used to predict T‐cell expansion and anti‐PD1 treatment response. These findings highlight a novel molecular mechanism indirectly regulating PD‐L1 through the STAT4‐related pathway: IL‐12R/JAK2–STAT3–STAT4/PD‐L1, and it has potential application in predicting anti‐PD‐1 immunotherapy response, which may pave the way for stratified immunotherapy in breast cancer.

## Graphical abstract

Schematic illustration of the prognostic role and gene function of STAT4 in breast cancer. Analyses of single‐cell datasets implies STAT4 is T‐cell specific in tumor environment. STAT4 upregulates PD‐L1 via STAT4‐related pathway: IL‐12R/JAK2–STAT3–STAT4/PD‐L1, which predicts treatment response in anti‐PD1 immunotherapy cohorts.

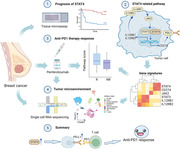

## 1 INTRODUCTION

Despite encouraging advances in the prevention and treatment of breast cancer in recent years, the incidence and mortality rates of breast cancer remain high.^1^ Breast cancer has surpassed lung cancer as the most common cancer diagnosed in women, accounting for 11.7% of the total number of patients diagnosed with cancer worldwide.^2^ Based on pathological features with human estrogen receptor (ER), progesterone receptor (PR), and epidermal growth factor 2 (HER2), breast cancer is categorized into three subtypes: luminal breast cancer (ER/PR+, HER2–), HER2‐positive breast cancer (HER2+), and triple‐negative breast cancer (TNBC) (ER–, HER2–, PR–).^3^ Another common classification of breast cancer based on gene expression is the intrinsic subtype of PAM50, including luminal A, luminal B, HER2 enriched, and Basal like.^4^ TNBC, with loss of ER, PR, and HER expression, is the most aggressive and easily metastatic subtype of breast cancer. Immunotherapy based on immune checkpoint blockade has shown that certain patients can benefit in TNBC.^5^

Signal transducer and activator of transcription 4 (STAT4), a crucial component of the STATs family, potentiates cell differentiation from native CD4+ T cell to T helper cell (Th1) via interleukin (IL)‐12/MKK6/p38α mediation and participates in the immune response through the production of interferon gamma (IFN‐γ) in tumor microenvironment (TME).^6^, ^7^, ^8^ Several cytokines activate STAT4 signaling, including IL‐12, IL‐23, IFN‐α, IL‐2, IL‐27, and IL‐35. IL‐12 is a crucial cytokine to trigger JAK2‐Tyk2 phosphorylation and subsequent STAT4 homodimerization. Afterwards, STAT4 promotes MYD88, IFN‐γ, Tumor necrosis factor (TNF), IL18R1, Furin, and IL18RAP transcription.^9^ STAT4 is also a key regulator of memory T‐cell differentiation, and the release of cytokines in natural killer (NK) cell, especially INF‐γ.^10^, ^11^ STAT4‐induced transcription factor T‐bet facilitates the expression of IFN‐γ, granzyme B, and perforin, resulting in enhanced NK cell cytotoxicity.^12^The signaling pathway of IL‐12/STAT4/IFN‐γ/TNF‐α in macrophage promoted anti‐PD1 therapy through macrophage CD8+ cytotoxic T‐lymphocyte (CTL) crosstalk.^13^

With the exception of STAT1 and STAT2, the other members of the STAT family have been shown to have a prognostic effect in breast cancer. STAT4 was found to be an excellent prognostic biomarker in HER2+ and Basal‐like subgroups of breast cancer cohort.^14^ In the TNBC subtype of “Immunity High,” STAT4 is one of the five core transcription factors.^15^ STAT4 is considered as CD8+ T related and favorable prognostic marker in gastric cancer.^16^ STAT4 was hypothetically considered as a TME‐related gene and prognostic factor of breast cancer.^17^ Similarly, among the STAT family members, those other than STAT2 and STAT3 have been confirmed as superb prognostic factors in ovarian cancer.^18^ STATs also perform potential roles in prognosis of glioma and diffuse gliomas.^19^, ^20^ Additionally, among 62 patients suffered gastric cancer, those with low STAT4 expression have poorer prognosis.^21^ STAT4 protein expression was significantly higher in pancreatic cancer than in non‐cancerous pancreatic tissue, and may serve as a biomarker for pancreatic cancer development and deterioration.^22^

However, the gene function and prognostic role of STAT4 in breast cancer remain poorly understood, particularly in TNBC. In this study, we explored the prognostic value and gene function of STAT4 in vitro and in vivo. Although single‐cell sequencing uncovered the heterogeneity of STAT4 expression in patients, STAT4 was specifically enriched in T cells in TME. Overexpression of STAT4 promoted programmed cell death ligand 1 (PD‐L1) level via the STAT4‐related pathway: IL‐12R/JAK2–STAT3–STAT4/PD‐L1 feedback axis in breast cancer cell. Furthermore, above pathway performed predictive effect to treatment response and T‐cell expansion in anti‐PD‐1 immunotherapy cohorts.

## 2 RESULTS

### 2.1 Prognostic role of STAT4 in independent cohort

**FIGURE 1:**
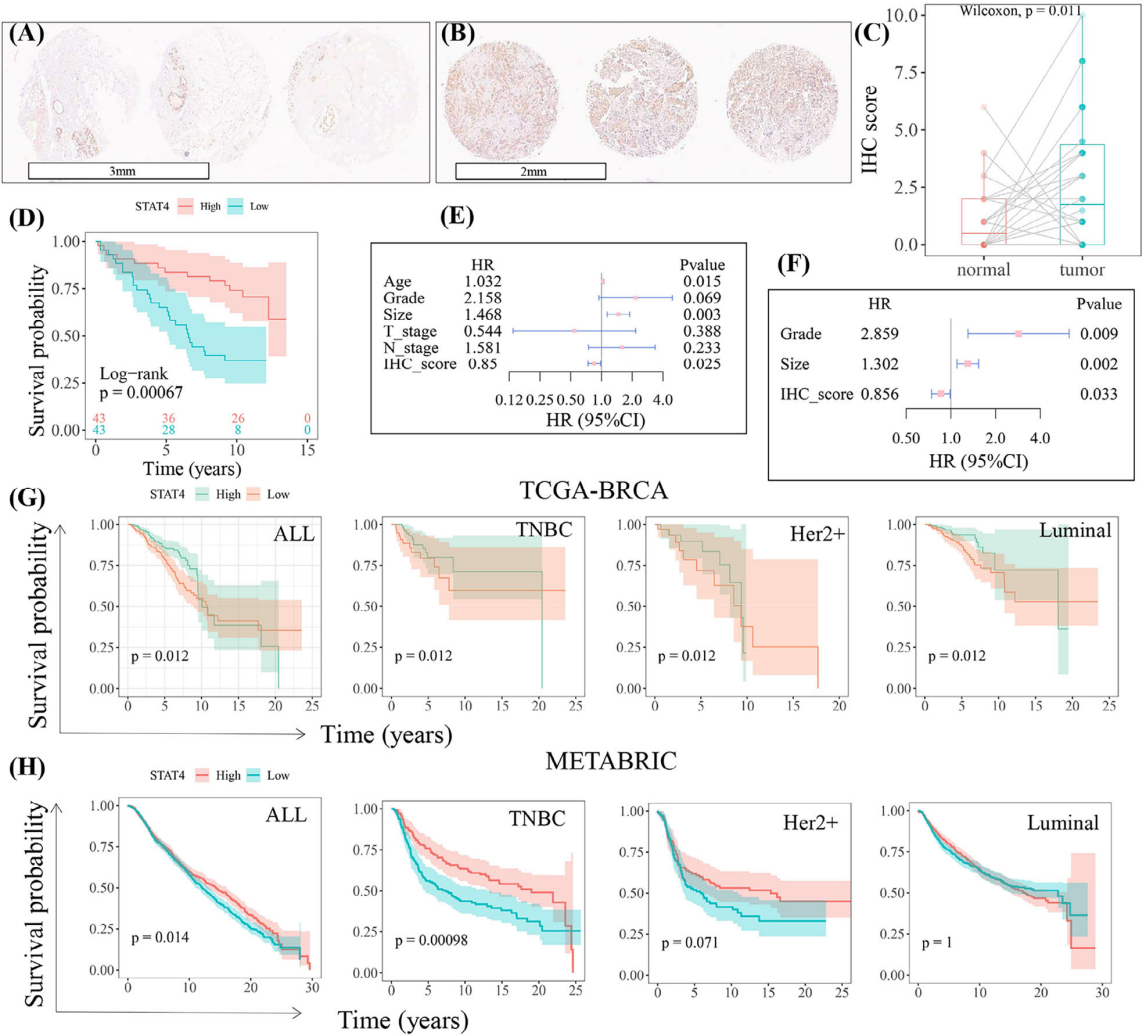
Diagnostic and prognostic values of signal transducer and activator of transcription 4 (STAT4) in breast cancer. Three representative immunohistochemical staining of STAT4 in tumor tissues (B) and matched normal tissues (A). The immunohistochemistry (IHC) score of STAT4 in triple‐negative breast tumor tissues (*N* = 86) and paired adjacent tissue (*N* = 45) (C). (D) Survival analysis of STAT4 in above collected cohort. (E and F) Forest plot showed the univariate COX analysis and multivariate COX analysis. (G and H) The survival analysis of STAT4 in The Cancer Genome Atlas (TCGA) breast cancer cohort (all subtypes, *N* = 905; TNBC, *N* = 184) and METABRIC dataset (all subtypes, *N* = 1903; TNBC, *N* = 298). 95% CI, 95% confidence interval; COX, Cox proportional hazard model; HR, hazard ratio; *N*, case numbers; TNBC, triple‐negative breast cancer.

Demonstrating the prognostic role of STAT4 in our collected cohort of 86 TNBC samples (Table S1), STAT4 protein was highly expressed in tumor tissues compared to that in paired normal tissues (Figure 1A–C), which was consistent with previous report.^23^ Survival analysis revealed high STAT4 expression was significantly related to better prognostic outcomes. Patients with high STAT4 expression showed longer survival time (Figure 1D). The univariate Cox proportional hazard assay implied clinical features age and tumor size were considered as risk factors of prognosis (hazard ratio > 1, *p* < 0.05), while the STAT4 score was a protective factor of prognosis (Figure 1E). Furthermore, significant univariate Cox characteristics were introduced into multivariate COX analysis, which identified STAT4 as an independent prognostic factor (Figure 1F). The Cancer Genome Atlas (TCGA) and METABRIC datasets were analyzed to further verify the prognostic value of STAT4 in a large cohort. As shown in Figure 1G,H, STAT4 was significantly associated with overall survival, and high STAT4 subgroups, particularly in TNBC, showed better prognostic survival.

### 2.2 STAT4 was specifically relevant with T cell in tumor microenvironment

**FIGURE 2:**
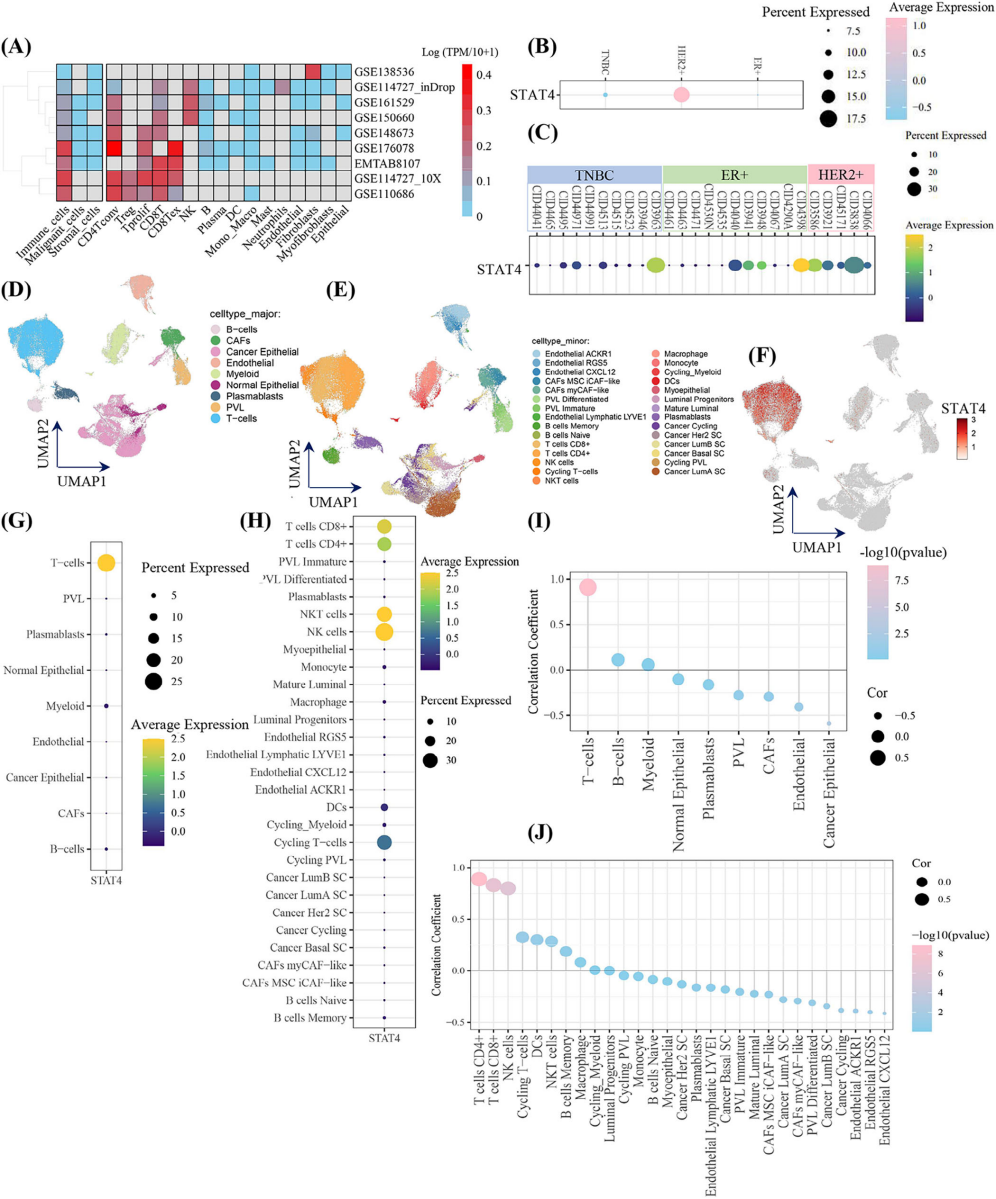
Single‐cell transcriptomics revealed the correlation between signal transducer and activator of transcription 4 (STAT4) and T cells in tumor microenvironment. (A) STAT4 expression levels in various single‐cell datasets (Tumor Immune Single‐Cell Hub 2 [TISCH2]) of breast cancer reveled by heatmap in TISCH2 database. Gray box indicates unavailable data. (B and C) GSE176078 (with 26 primary tumors of breast cancer, including estrogen receptor [ER+], epidermal growth factor [HER2+], triple‐negative breast cancer [TNBC] patients) was used to demonstrate the heterogeneity of STAT4 expression in breast cancer subtype and in each patient. (D and E) The dimensional Uniform Manifold Approximation and Projection (UMAP) drawing major and minor cell clusters in GSE176078 cohort. (F–H) The STAT4 expression at single‐cell level analyzed by UMAP plot and dot plots. (I) The correlation between STAT4+ cell ratio and the ratio of major cell types in patients. The correlation toward T cells was specifically shown. (J) The correlation between STAT4+ cell ratio and ratio of 29 minor cell types. B, B cells; CAF, cancer‐associated fibroblasts; CD4TCONV, conventional CD4+ T cells; CD8T, CD8+ T cells; CD8Tex, exhausted CD8 T cells; Cor, Pearson's correlation coefficients; DC, dendritic cells; Mono_Macro, monocytes or macrophages; NK, natural killer cells; PVL, perivascular‐like cells; Tprolif, proliferating T cells; Treg, regulatory T cells.

To further decipher the role of STAT4 in TME, single‐cell transcriptomics datasets of nine breast cancer cohorts were analyzed. As presented in Figure 2A, a distinct expression pattern was discovered among various datasets, with different expression level and distribution in cell clusters were observed, unveiling the expression heterogeneity of STAT4 among patient cohort. STAT4 was predominantly expressed in immune cells but not in malignant cells and stromal cells. Detailed cell annotations suggested STAT4 was high expression in CD4+ T cells, proliferating T cells, and CD8+ T cells in more than half the data sets (Figure 2A). The single‐cell sequencing dataset of clinical cohort GSE176078 (a comprehensive dataset comprising 26 primary breast cancer tumors) was selected to further explore STAT4 in TME. In breast cancer subtypes, STAT4 was higher in HER2+ samples than in ER+ and TNBC patients (Figure 2B). Moreover, the expression heterogeneity of STAT4 in patients was reveled in Figure 2C. Importantly, the elevated and specific expression of STAT4 was observed in T cells (Figure 2D–F), and further minor annotations implied that STAT4 was expressed in multiple anti‐tumor T‐cell subtype: CD4+ T cells, CD8+ T cells, NK T cells, NK cells, and cycling T cells (Figure 2G,H). The significant and positive correlation (Cor = 0.91, *p*‐value < 0.001) between T‐cell ratio and STAT4‐positive cell ratio in the 46 primary tumor samples was shown (Figure 2I). More detailed annotations suggested STAT4+ cells were strongly correlated (Cor > 0.8, *p*‐value < 0.001) with CD4+ T cells, CD8+ T cells, and NK cells (Figure 2J), having implications for understanding favorable prognosis in the STAT4‐high cohort. Briefly, high‐STAT4 cohort exhibited higher T‐cell level, resulting in better prognosis status.

### 2.3 STAT4 as a tumor driver in vitro

**FIGURE 3:**
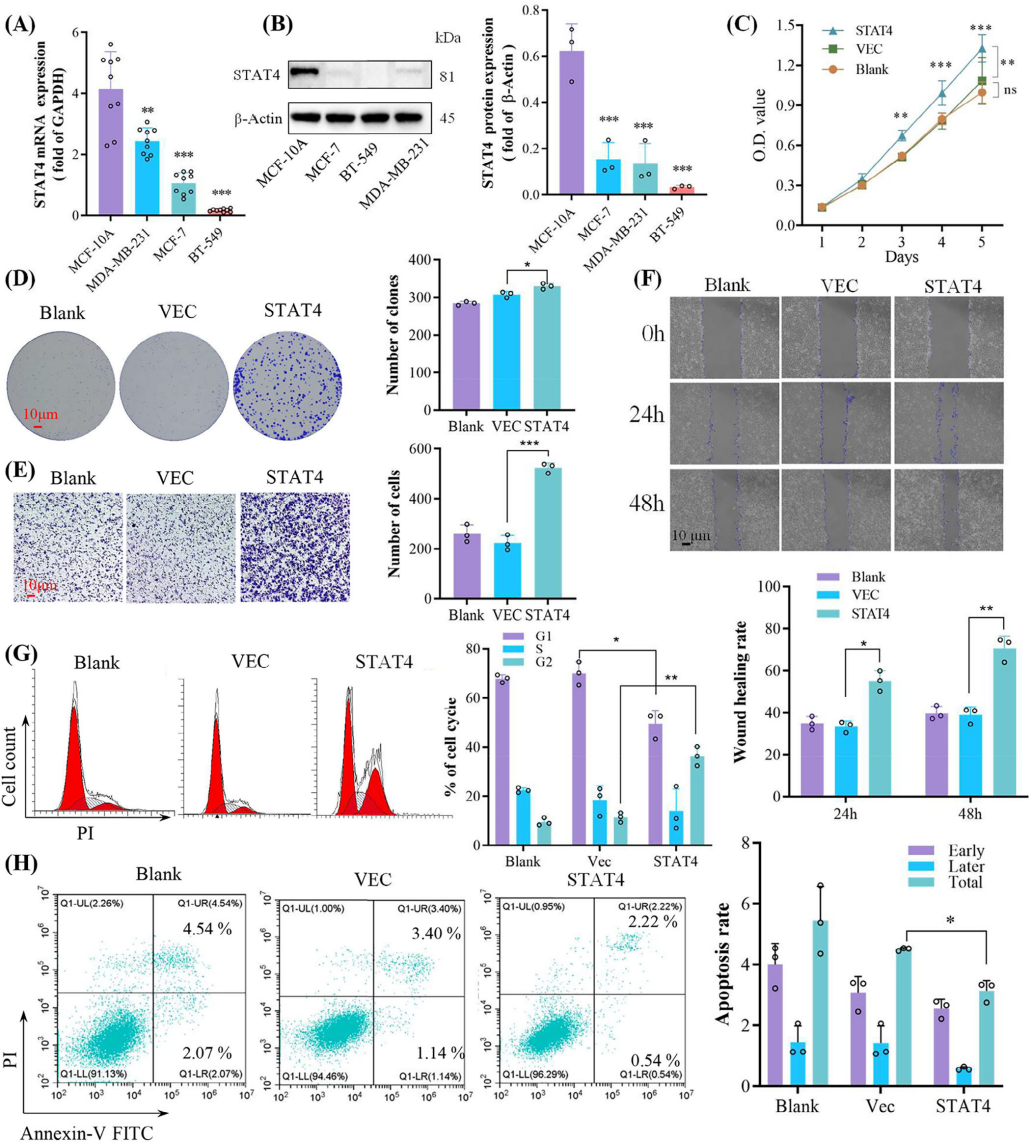
Overexpressing of signal transducer and activator of transcription 4 (STAT4) reduced apoptosis while promoting cell proliferation, migration, invasion and cell cycle. The mRNA (A) and protein (B) expression of STAT4 in human mammary epithelial cells MCF‐10A and breast cancer cells MDA‐MB‐231, BT‐549, and MCF‐7. (C) Overexpression of STAT4 promoted BT549 cell proliferation. The experimental results of clone formation (D), Transwell (E), and wound healing (F) assays. Cell cycle (G) and apoptosis analysis (H) after transfection for 24 h. O.D. value, optical density. Biological repeat, *N* = 3.

To unveil gene function of STAT4 in breast cancer cell lines in vitro, we first detected the mRNA and protein expression of STAT4 in several common breast cancer cell lines. Almost no STAT4 expression is detected in three of breast cancer cell lines, but MCF‐10A cells display high levels of STAT4 expression (Figure 3A,B). STAT4 gene was overexpressed by plasmid delivery into BT549 cells. Overexpression of STAT4 promoted BT549 cell proliferation revealed by cell counting kit‐8 (CCK‐8) and cell clone assays (Figure 3C,D). Wound healing and Transwell experiments implied that STAT4 facilitated migration and invasion (Figure 3E,F). Simultaneously, STAT4 overexpression led to a shortening of the G1 cell cycle and a decrease in the proportion of apoptotic cells (Figure 3G,H).

### 2.4 STAT4 upregulated PD‐L1 via IL‐12R/JAK2–STAT3–STAT4/PD‐L1 axis

**FIGURE 4:**
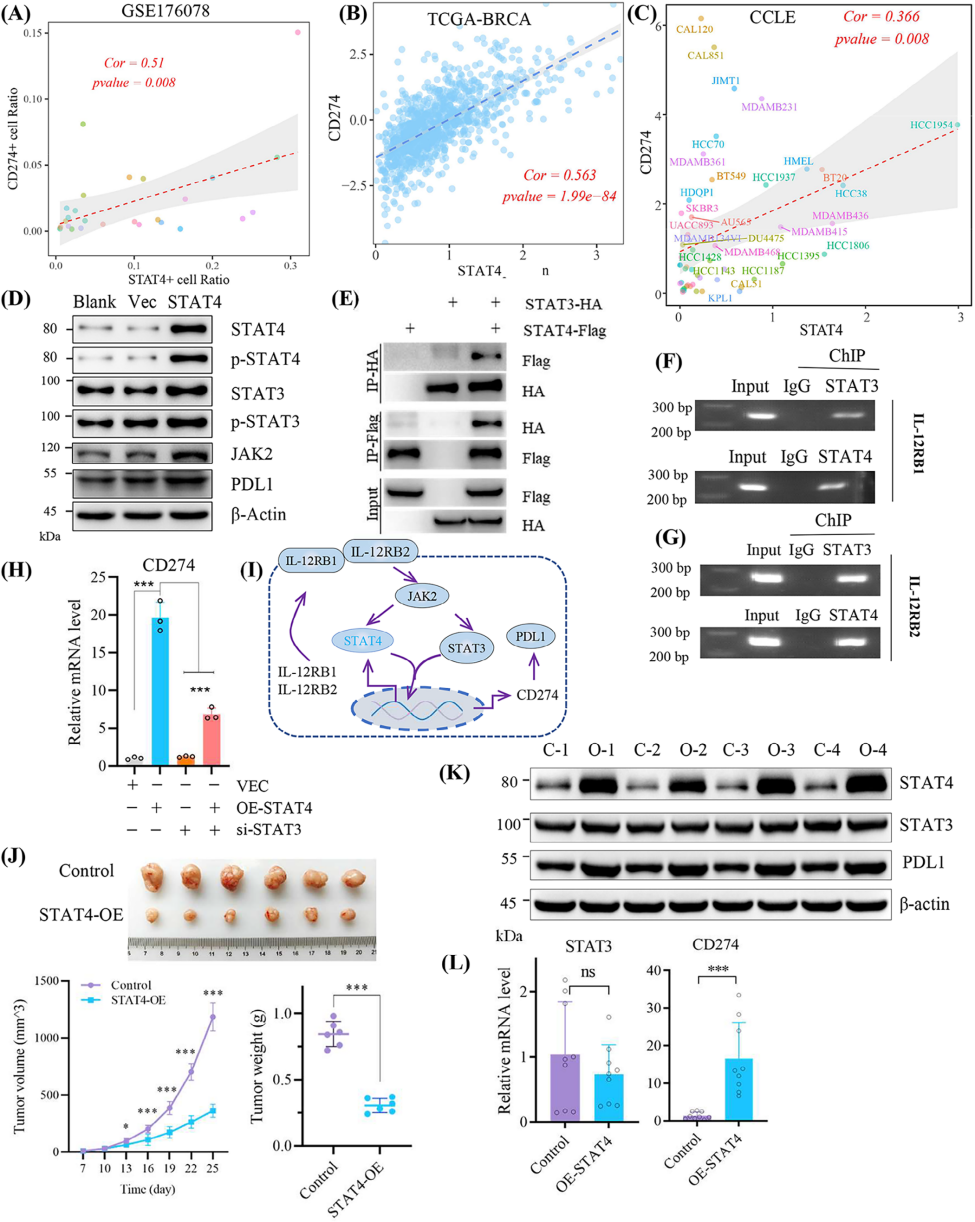
Signal transducer and activator of transcription 4 (STAT4) upregulated programmed cell death ligand 1 (PD‐L1) via the IL‐12R/JAK2–STAT3–STAT4/PD‐L1 axis. (A and B) The correlation between CD274 (coding PD‐L1 protein) and STAT4 in clinical cohort (GSE176078 and The Cancer Genome Atlas [TCGA]–breast invasive carcinoma [BRCA]), and in 59 breast cancer cells of Cancer Cell Line Encyclopedia database (C). (D) Overexpressed STAT4 facilitated JAK2/STAT3/PD‐L1 expression, as revealed by western blotting. (E) Co‐immunoprecipitation (Co‐IP) assay indicated direct interaction between STAT3 and STAT4. (F and G) Chromatin immunoprecipitation (ChIP) analysis verified STAT3 and STAT4 synergistically transactivated IL‐12RB in HEK‐293T cells. (H) Reverse transcription‐quantitative polymerase chain reaction (RT‐qPCR) assay relative mRNA expression in vector, overexpressed STAT4 and si‐STAT3 subgroups. (I) Graphic illustration displaying the model that STAT4 regulated PD‐L1 expression dependent on JAK2/STAT3 axis. (J) The nude mice experiment of bearing xenograft tumor in control (vector) and STAT4‐OE subgroups. (K) Protein expression of STAT3, STAT4, and PD‐L1 in nude mice tumors (C, control; O, STAT4‐OE). (L) Relative mRNA expression in control and OE‐STAT4 subgroups (three mice per group, *N* = 3).

TNBC cell lines expressed PD‐L1,^24^ and immune checkpoint inhibitors have shown great treatment potential in TNBC.^25^ Interestingly, the co‐transcription between STAT3 and STAT4 has been reported in colorectal carcinoma,^26^ but whether STAT4 indirectly transactivates CD274 (coding PD‐L1) through synergistic effect with STAT3 remains unknown. Therefore, we explored the interaction between STAT4 and JAK2/STAT3/PD‐L1 axis. A strong correlation between STAT4 and CD274 was unmasked in clinical single‐cell dataset GSE176078 and TCGA–breast invasive carcinoma (BRCA) cohort, and in breast cancer cell lines (Figure 4A–C). Herein, STAT4 overexpression promoted PD‐L1 expression and JAK2/STAT3 overactivation (Figure 4D), whereas the possibility that STAT4 transcriptionally induced PD‐L1 expression with STAT3 dependent was not excluded.^27^, ^28^ Previous research reported the close correlation between IL12RB1 and the activation of STAT3 and STAT4, while IL12RB2 specifically activated STAT3.^29^ In this study, co‐immunoprecipitation assay verified the direct binding between STAT3 and STAT4 (Figure 4E). Both STAT3 and STAT4 were responsible for the transcription of IL12RB1 and IL12RB2 in breast cancer cell (Figure 4F,G). Silencing STAT3 reduced CD274 level induced by STAT4 overexpression, indicating that STAT4 regulated PD‐L1 expression dependent on STAT3 (Figure 4H). The above results implied that STAT4 promoted PD‐L1 expression through the JAK2/STAT3/IL12RB axis, thus we proposed the potential feedback loop: STAT4/IL12RB/JAK2–STAT3–STAT4/PD‐L1 pathway (Figure 4I). Interestingly, in xenograft nude mice model, overexpressed STAT4 decreased tumor growth (Figure 4J). Overexpressed STAT4 elevated PD‐L1 expression in protein and mRNA level in vivo, instead of STAT3 (Figure 4K,L). These results suggested that STAT4 enhanced cell proliferation in a STAT3‐dependent manner in vitro, while STAT4 inhibited tumor growth without STAT3 dependent in vivo.

### 2.5 STAT4‐related pathway score predicted anti‐PD‐1 immunotherapy response

**FIGURE 5:**
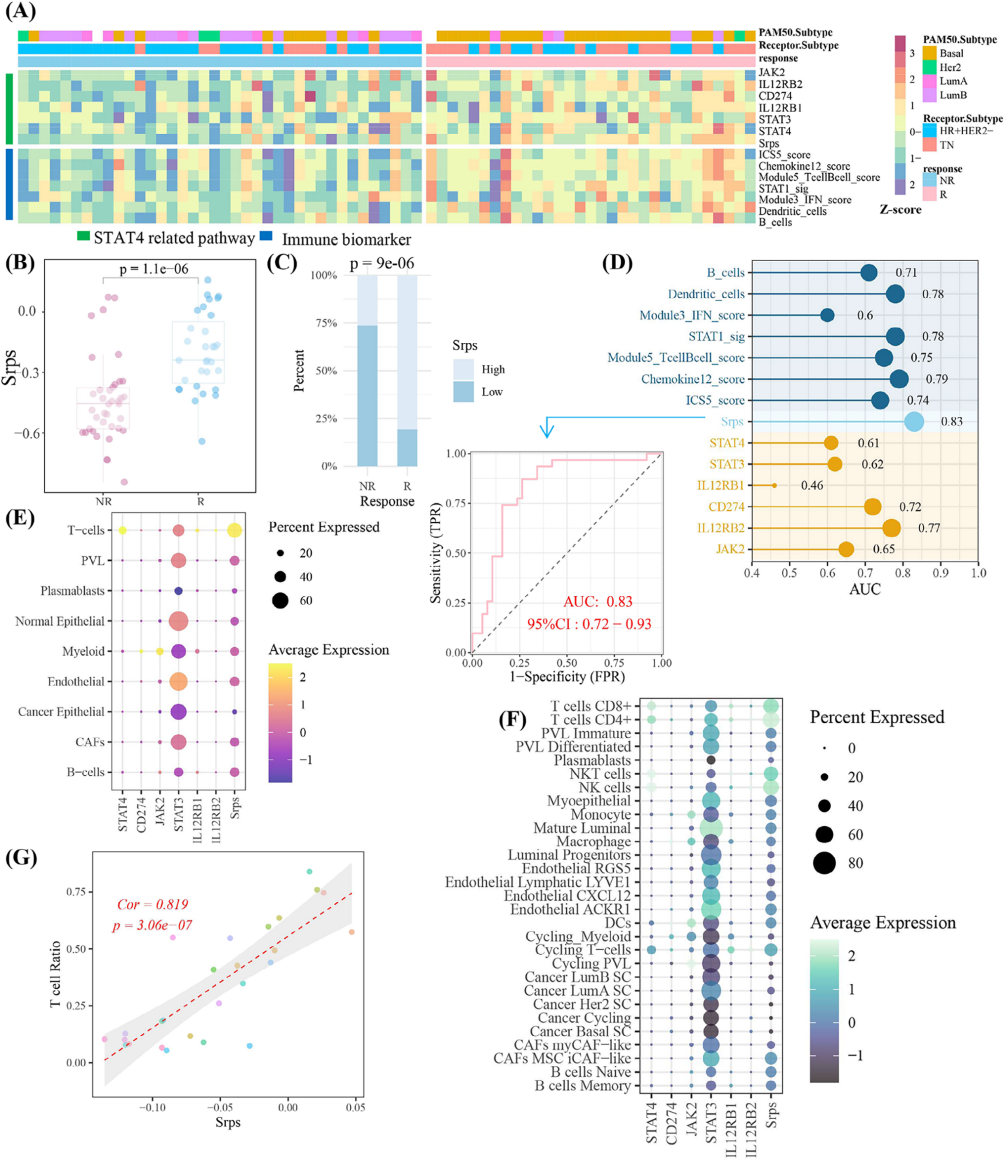
Signal transducer and activator of transcription 4 (STAT4)‐related pathway score associated with T cells predicted anti‐PD1 immunotherapy response in the GSE194040 cohort (Pembrolizumab, anti‐PD1). (A) The expression landscape of STAT4‐related pathway members and seven immune biomarkers. (B) Srps was significantly varied among responders and non‐responders. (C) The percent of responders and non‐responders in Srps subgroups. Significant difference in categorical variables was examined using Fisher's exact test. (D) The area under the curve (AUC) of receiver operating characteristic curve for prediction performance (AUC = 0.83, sensitivity = 0.87, specificity = 0.74). (E and F) The expression pattern of numbers of STAT4‐related pathway and Srps in tumor microenvironment of GSE176078. (G) The correlation between T‐cell ratio and Srps score. CAF, cancer‐associated fibroblasts; Cor, Pearson's correlation coefficients; NR, non‐responder; *p*, *p*‐value; PVL, perivascular‐like cells; R, responder; TN, triple‐negative breast cancer (TNBC).

Given that STAT4 elevated PD‐L1 expression via the feedback loop: STAT4/IL‐12R/JAK2–STAT3–STAT4, in order to further apply above signaling axis for predicting immunotherapy response, the feedback loop was defined as the STAT4‐related pathway. The STAT4‐related pathway score (Srps) was calculated using its gene symbols (JAK2, STAT3, STAT4, CD274, IL12RB1, IL12RB2) in GSE194040 cohort. The higher expression of components of STAT4‐related pathway and Srps in the responder cohort than in non‐responder cases was observed, respectively, with a consistent trend among seven immune activation biomarkers^30^ (Figure 5A), suggesting high‐Srps cases had more “hot” tumor environment than patients with low Srps levels.^31^ As shown in Figure 5B,C, the Srps was significantly elevated after anti‐PD1 treatment, and responders were more frequent in the high‐Srps subgroup. The predictive accuracy of Srps (area under the curve [AUC] = 0.83) toward treatment response was better than that of seven biomarkers (Figure 5D). In single‐cell cohort of GSE176078, STAT4 and Srps were mainly expressed in T cells, comprising CD4+ T cells, CD8+ T cells, NK cells, and NK T cells, and Srps was strongly associated with T‐cell levels (Figure 5E–G). These findings highlighted Srps as a T‐cell‐related signature for immunostratified therapy, with potential to predict anti‐PD‐1 treatment response.

### 2.6 The immune landscape of STAT4‐related pathway in anti‐PD1 immunotherapy

**FIGURE 6:**
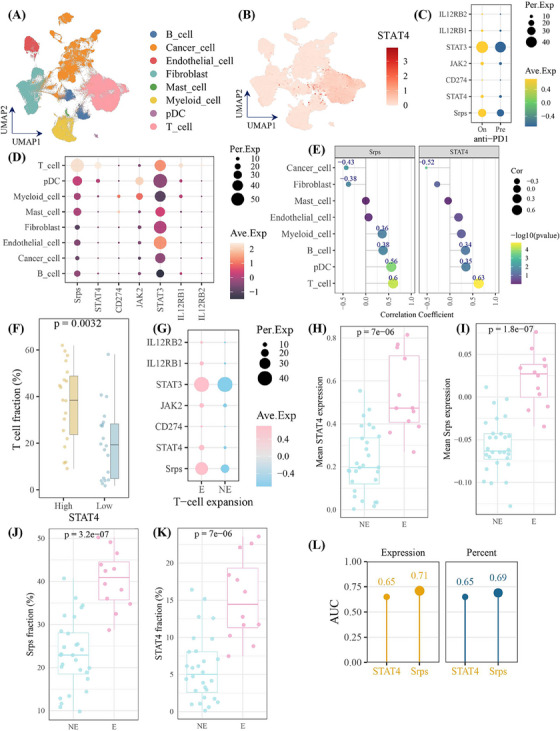
Single‐cell transcriptomic revealed signal transducer and activator of transcription 4 (STAT4)‐related pathway was upregulated and predicted T‐cell expansion during anti‐PD1 immunotherapy. (A and B) The dimensional Uniform Manifold Approximation and Projection (UMAP) showing cell type distribution and STAT4 expression in the anti‐PD1 single‐cell cohort. (C) Dotplot showing the expression of Srps and STAT4‐associated pathway members after anti‐PD1 treatment (On) or before anti‐PD‐1 treatment (Pre). (D) Expression of Srps and STAT4‐associated pathway members in cell types. (E) Relationship between Srps or STAT4 and cell subtypes. Significant results are indicated by number with navy color. (F) T‐cell percentage in STAT4 subgroups. (G) Dotplot showing the expression of Srps and STAT4‐associated pathway members in patients with (E) or without T‐cell expansion (NE). (H–K) The expression value and expression proportion of STAT4 and Srps in patients, respectively. (L) The area under the curve (AUC) of receiver operating characteristic curve of STAT4 and Srps in pre‐treatment to predict T‐cell expansion after anti‐PD‐1 treatment. Ave. Cor, correlation coefficient; Exp, average expression; pDC, plasmacytoid dendritic cell; Per.Exp, percent expression.

To further investigate the role of STAT4‐related pathway in tumor environment following anti‐PD1 treatment, we analyzed the single‐cell dataset of breast cancers treated with anti‐PD‐1 immunotherapy (cases = 29). As present in Figure 6A,B, STAT4 was high expression in T cells. Of importance, the STAT4‐related pathway members and Srps were increased following anti‐PD1 treatment (Figure 6C). Notably, STAT4 and Srps were enriched in T cells, and exhibited strong correlation with T‐cell (Figure 6D,E), verifying their robust expression pattern in T cell with or without anti‐PD1 treatment, and strong relationship with T cell (Figure 6E–G). Moreover, STAT4‐high subgroup had significantly greater T‐cell infiltrating level than STAT4‐low subgroup (Figure 6F). Furthermore, the expression level and expression percent of STAT4 and Srps were significantly increased in T‐cell expansion patients (Figure 6G–K). It is worthy to note that Srps level in pre‐treatment samples could predict T‐cell status (expansion vs. non‐expansion) after anti‐PD‐1 with available accuracy (Figure 6L). Collectively, Srps signatures showed predictive potential for T‐cell expansion following anti‐PD1 treatment. We also applied the Srps on large‐scale dataset with anti‐PD‐1/PD‐L1/CTLA4 treatment and found that Srps performed certain accuracy to predict treatment response on eight of total 25 cohorts (AUC > 0.7), which demonstrated the potential utility of Srps in pan‐cancer immunotherapy response (Figure S1).

## 3 DISCUSSION

A previous study showed that STAT4 overexpression promoted ovarian cancer metastasis to trigger the activation of cancer associated fibroblasts and the development of epithelial–mesenchymal transition (EMT), which was mediated via Wnt7a.^32^ In another study, STAT4 protein overexpression induced by hypoxia accelerated the progression of EMT in ovarian cancer cells, which was negatively regulated by miR‐200a via targeting STAT4. Interestingly, there was no significant difference of STAT4 in transcription level between normoxia and hypoxia.^33^ Conversely, STAT4 protein was expressed at lower levels in liver cancer tissues than normal tissues. Defective STAT4‐induced lymphatic metastasis and immune suppression via increased lymph‐angiogenesis with elevated VEGFA expression and decreased production of IFN‐γ with CD4^+^ cell dependent in STAT4 deficient tumor bearing mice. The accumulation of MDSCs and systemic depletion of Th17 cells and IL‐17 were also attributed to STAT4 deficiency in head and neck squamous cell carcinoma.^34^ High expression of STAT4 was found in colorectal cancer tissues, compared to adjacent tissues and silencing of STAT4 resulted in the weakening of colorectal cancer proliferation and invasion.^35^ Similar results have been reported in bladder cancer, where the expression of STAT4 in cancerous tissues was higher than that in adjacent non‐cancerous tissues at both the protein and mRNA level. Decreased STAT4‐induced proliferation inhibition and apoptosis in sw780 and T24 cells, however, co‐transfection of mir‐200a‐3p mimics and STAT4 reversed this effect.^36^ In contrast, STAT4 showed the tumor suppressor effect in hepatocellular carcinoma (HCC) and was significantly correlated with Ki67 expression, histological grade, HBV infection, and tumor size in HCC clinical cohort.^37^

Here, we revealed the prognostic value of STAT4 in clinical cohorts of breast cancer and demonstrated the oncogenic role of STAT4 in breast cancer BT549 cells. Intriguingly, STAT4 upregulated PD‐L1 expression was observed, and was dependent on the JAK2/STAT3 signaling axis. Furthermore, a positive feedback loop: IL‐12R/JAK2–STAT3–STAT4/PD‐L1 was also proposed and defined as the STAT4‐related pathway, whose score we applied to predict anti‐PD‐1 immunotherapy response. These results also elucidated an indirect regulatory strategy to modulate PD‐L1 expression through collaborative effect with its transcription factor.^27^

Although exogenous overexpression of STAT4 showed oncogenic effect in vitro, STAT4 specific expression in T cells and its positive correlation with T‐cell infiltration level might be responsible for better prognosis in STAT4‐high cohort. Moreover, STAT4 promoted anti‐tumor immune responses comprised Th1, Th17, and IFN‐γ production in HNSCC.^34^ STAT4 also facilitated NK cell cytotoxicity, proliferation and differentiation of Th1 cells, and stimulation of IFN‐γ‐mediated macrophage T‐cell crosstalk.^13^, ^34^ STAT4 is positively correlated with IFN‐γ in the breast cancer TME with or without anti‐PD‐1 treatment (Figure S2). Here, STAT4 overexpression inhibited tumor growth without STAT3 dependent in nude mice model. Thus, STAT4 may hasten Th1 differentiation, T‐cell infiltration, and IFN‐γ response, possibly leading to favorable survival in patients.

PD‐L1 transactivated immune‐response‐related genes including IFN‐γ‐mediated signaling and major histocompatibility complex class I (MHC‐I) antigen presentation, resulting in treatment sensitivity to PD‐1 blockade.^24^ Immune induction may turn cold breast tumor hot to approach threshold of PD‐L1 in the treatment of breast cancer, and anti‐PD1 therapy has been applied to TNBC.^38^, ^39^ JAK2/STAT3 signal activation benefits treatment response in PD‐1 blockade therapy after immune induction.^40^ In our study, STAT4 upregulated PD‐L1 expression via the STAT4‐related pathway and elevated MHC‐II (Figure S3), which presumably contributed to favorable prognosis and anti‐PD1 response though enhancing the IFN pathway and MHC‐mediated neoantigen presentation to T cells. Recently, STAT4 indirectly modulated MHC‐I neoantigen presentation and CTL‐mediated anti‐tumor effect via KDM5D–AP1/2–MHC‐I pathway in colon cancer,^41^ however this mechanism is still unexplored in breast cancer (Figure S3).

A potential strategy to activate STAT4 to promote macrophage‐cytotoxic T‐cell interaction in breast cancer tumor environment seems promising.^13^ However, further evidences are needed in STAT4‐high expression cell lines and genome editing animal models to fully understand the role of STAT4 in breast cancer. Overexpression or agonist of STAT4 may be employed to induction favorable TME for cancer immunotherapy.^42^ Collectively, our findings provide a potential choice for immune induction dependent on the STAT4‐related pathway: IL‐12R/JAK2–STAT3–STAT4/PD‐L1 that provides potential clinical implications for predicting anti‐PD‐1 immunotherapy response.

## 4 MATERIAL AND METHODS

### 4.1 Data source and processing

The mRNA sequencing data of BRCA with fragments per kilobase per million (FPKM) files were retrieved from TCGA (https://portal.gdc.cancer.gov/) and normalized by log2(FPKM +1) for further analysis (113 normal and 1109 tumor cases) in R software (version 4.0). The gene expression and clinical data of Molecular Taxonomy of Breast Cancer International Consortium (METABRIC, 1903 cases) cohort were retrieved from cbioportal (http://www.cbioportal.org/). The gene expression matrix and annotations of 1019 cell lines (more than 20 cancer types, including 59 breast cancer cell lines) were download from the Cancer Cell Line Encyclopedia (https://sites.broadinstitute.org/ccle) and then normalized by above log2 method. Duplicate samples and those without prognostic information were excluded from further analysis. The 86 breast cancer samples with clinicopathological data were collected for discovering protein expression and prognosis in breast cancer (Table S1). The STAT4 expression in nine single‐cell transcriptomic datasets of human breast cancer was deciphered in Tumor Immune Single‐Cell Hub 2 online server (http://tisch.comp‐genomics.org/). Single‐cell dataset of 26 primary breast tumors (including 11 ER+, five HER2+, and 10 TNBC samples) was download from Gene Expression Omnibus (GEO, https://www.ncbi.nlm.nih.gov/geo/) under the accession number GSE176078 and processed following previous study.^43^ The bulk transcriptomic dataset of PD1 immunotherapy trail (pembrolizumab, 69 cases) was retrieved and refined from GEO database (GSE194040). The single‐cell dataset with anti‐PD1 treatment (pembrolizumab, 29 cases) in breast cancer was retrieved from previous study.^44^ The data of immunotherapy on pan‐cancer were analyzed in TIDE (http://tide.dfci.harvard.edu).^45^ The STAT4‐related pathway score (gene set: JAK2, STAT3, STAT4, CD274, IL12RB1, IL12RB2) was computed using ssGSEA method of GSEA package in R software (version 4.3.0).^46^ The mean expression in *Z*‐score was shown in heat map, and dot plot was used to exhibit gene expression at single‐cell level.

### 4.2 Expression and prognosis of STAT4 in BRCA

The median value of STAT4 expression level was used as the cut‐off to distinguish between the high and low expression groups. The significance of the Kaplan–Meier survival curve was obtained from log‐rank test. The tissues microarrays (TMA) ship of 86 TNBC samples and 45 paired tumor adjacent tissues were collected from National Human Genetic Resources Sharing Service Platform (NHGRP; ID: 2005DKA21300). The subsequent experiments were performed at Shanghai Outdo Biotech Co., Ltd. and approved by the company's ethics committee (no. HBreD139Su01; HBre‐Duc090Sur‐01). TMAs were constructed and fixed with formalin. The staining of immunohistochemistry (IHC) was completed by auto‐stainer link 48 instruments (Dako) with STAT4 antibody (1:500, Abcam, ab68156) overnight at 4°C. IHC results were scanned by Ape‐rio XT (Leica Microsystems Inc.) and evaluated by two pathologists independently via modified immunoreactive score (IRS). The staining intensity of negative, weak, moderate and strong were scored as 0, 1, 2 and 3, respectively. The percentage scores of positive cells were described as 0, 0%; 1, 1%–25%; 2, 26%–50%; 3, 51%–75% and 4, 76%–100%. The collected clinical cohort was divided into STAT4‐high and STAT4‐low subgroups according to the median of IHC scores, and table was constructed by R package TableOne with the default significance test method for categorical variables: Fisher's exact test.

### 4.3 Cell culture and cell transfection

MCF‐7, MCF‐10A, MDA‐MB‐231, and BT549 cells were purchased from the Cell Bank of the Chinese Academy of Sciences. With the exception of MCF‐10A, cell lines were cultured in RPMI 1640 medium (Gibco) supplemented with 10% fetal bovine serum (FBS, Gibco; Thermo Fisher Scientific) and 1% penicillin and streptomycin solution (HyClone, Cytiva), while the MCF‐10A cells were incubated with DMEM‐F12 (Gibco) with 10% FBS, 0.3 g/L L‐glutamine, 20 ng/mL Epidermal growth factor (EGF), 10 μg/mL insulin, 500 μg/mL hydrocortisone, and 40 mg/L gentamicin 23. All cells were maintained under environmental conditions of 37°C and 5% CO_2_. The amplified cDNA of human STAT4 was cloned into the pLenti‐CMV‐EGFP lentiviral vector to construct the STAT4 overexpression vector (Gene Pharma). After 6 h of transfection with polyethylenimine agent for si‐STAT3 (sense: 5′‐ GGCCAGCAAAGAAUCACAUTT‐3′, antisense: 5′‐AUGUGAUUCUUUGCUGGCCTT‐3′), the medium was transferred to complete medium.

### 4.4 Western blotting and co‐immunoprecipitation assay

For western blotting, proteins were extracted from the cells using RIPA lysis buffer (Beyotime, P0013). The protein concentration was measured using bicinchoninic acid protein assay kit (Beyotime, P0009). Denatured total protein was loaded onto 10% SDS‐PAGE gel and then transferred to the polyvinylidene fluoride (PVDF) membrane. The blots were incubated with monoclonal antibodies (STAT4, 2653, CST; p‐STAT4, 4134, CST; PD‐L1, 13684, CST; STAT3, 4904, CST; p‐STAT3, 9145, CST; JAK2, 3230, CST; β‐actin, AC026, ABclonal), and then secondary horseradish peroxidase (HRP)‐conjugated anti‐rabbit antibody (ab6721, Abcam) was added. After washing, membranes were subjected to chemiluminescence, and the relative optical density was analyzed using ImageJ software (NIH).

Immunoprecipitation: after transfection, cells were lysed in Pierce IP Lysis Buffer (Thermo Fisher Scientific) and the supernatants were cultured overnight with anti‐Flag (ab205606, Abcam) or anti‐HA (ab236632, Abcam) antibody. Immunoprecipitation reaction mixtures were incubated with protein A/G beads (Santa Cruz Biotechnology). The beads were washed three times to elute the proteins before performing western blotting as described above.

### 4.5 Reverse transcription‐quantitative polymerase chain reaction and chromatin immunoprecipitation

Reverse transcription‐quantitative polymerase chain reaction (RT‐qPCR): total RNA was extracted using TRIzol reagent. After 24 h of cell inoculation, reverse transcription was performed to synthesize cDNA through the Primescript RT Reagent Kit (Takara Bio Inc., RR047A). The qPCR analysis was performed using quantum studio 3 (Thermo Fisher Scientific) based on SYBR kit TB green TM premix ex taqtm II (Takara Bio Inc., RR820A). Thermal cycling conditions were set according to the manufacturer's instructions, and the primer sequences were as follows: ACTB—forward primer: CGATCCGCGCGTCCCACA and reverse primer: ACGCAGCTCATTGTAGAAGGGTGGTG; STAT4—forward primer: AGCCTTGCGAAGTTTCAAGA and reverse primer: ACACCGCATACACACTTGGA; CD274—forward primer: GCCTCCACTCAATGCCTCAATTTG and reverse primer: TTCACAACCACACTCACATGACAAG. Relative mRNA expression levels were measured via the 2^− ΔΔCT^ method.

Chromatin immunoprecipitation (ChIP) analysis was carried out using the Pierce Agarose ChIP Kit (Thermo Fisher Scientific, 26156), according to the manufacturer's instructions. In brief, cross‐linked protein–DNA complexes were immunoprecipitated by following antibodies: anti‐STAT3 (Santa Cruz Biotechnology, sc‐8019), anti‐STAT4 (Santa Cruz Biotechnology, sc‐398228), or negative control IgG (Abcam, ab172730). PCR reactions (Takara, RR900Q) electrophoresis were used to locate the target gene promoter sequences in the immunoprecipitated DNA. The sequences of the PCR primers for IL12RB promoter are as follows: IL12RB1—forward primer: GCTGGAGTGTGGTGGCATGATC and reverse primer: AGGTGGGTGGATCACTTAGGGTTAG; IL12RB2—forward primer: GTAATGCCCAGAAGCGTGGT and reverse primer: CTGCCAGGTAAACAATAACAATGC.

### 4.6 Cell proliferation assay

After transfection of the three groups (blank control, empty vector, and STAT4 overexpression), cells were harvested and seeded into 96‐well plates at a density of 2000 cells/well. After 24, 48, 96, and 120 h, 10 μL of CCK‐8 (CK04, Dojindo) reagent was added to each well according to the manufacturer's instructions and the cells were incubated at 37°C for 1 h. The absorbance at 450 nm was obtained using microplate reader (Thermo Fisher Scientific).

### 4.7 Wound healing and Transwell analysis

BT‐549 cells were seeded in six‐well plates and cultured to approximately 100% confluence in each well. Linear scratch was generated via 10‐μL pipette tips. Photographs of scratches were taken after 24 and 48 h for generating statistics through ImageJ software. In Transwell assay, 1 × 10^5^ transfected cells were suspended in 200 μL of serum‐free medium and were transplanted to the upper Transwell chamber (Corning, Inc., 0.8 μm pore) covered with Matrigel (Corning, Inc.).^47^ Complete medium of RPMI 1640 (500 μL) was added to the lower chamber. After 24 h, the cells were washed with phosphate‐buffered saline buffer, fixed with 4% paraformaldehyde, stained with 0.1% crystal violet and counted via ImageJ.

### 4.8 Flow cytometry assay

BT‐549 cells (2 × 10^5^ cells/well) were seeded into six‐well plates for cell cycle analysis and apoptosis assay. Transfected cells were fixed by 70% ethanol overnight at −20°C before staining with propidium iodide (PI) cycle kit (Nanjing KeyGen Biotech. Co. Ltd.) for cell cycle analysis. For apoptosis analysis, transfected cells were collected and stained with annexin V/PI apoptosis detection kit (KeyGen Biotech). The flow cytometry analysis was exerted using flow cytometer (CytoFlex, Beckman Coulter), and the MODIFIT LT 5.0 (BD Biosciences) was used for data analysis of the cell cycle assay.

### 4.9 Animal experiment

BALB/c nude mice were purchased from SPF Biotechnology Co., Ltd.. Animal experiments were performed and approved at the Animal Central of Chengdu University of Traditional Chinese Medicine. Female mice were grouped randomly and subcutaneously injected with wild‐type BT549 cells or STAT4‐overexpressed BT549 cells (5 × 10^6^ cells/mouse) to establish a cell‐derived xenograft model. Animals were caged in a specific pathogen‐free laboratory with free access to food and water for 25 days. After the animals were euthanized by cervical dislocation, tumor weight, and tumor volume were measured. Tumor tissues were used to perform qPCR and western blot assays.

### 4.10 Statistical analysis

All statistical analyses of public database were processed and visualized in R software. Statistical significance was calculated using Wilcoxon signed rank test and Kruskal–Wallis test in two or more groups, respectively. All correlation were assessed by Pearson's method. The experiments were repeated thrice in parallel, and GraphPad PRISM software (version 7.0) was applied to calculate the significance of all experiments. The *p*‐value of less than 0.05 was considered statistically significant (ns, *p* ≥ 0.05; ^*^ *p* < 0.05; ^**^ *p* < 0.01; ^***^ *p* < 0.001).

## AUTHOR CONTRIBUTIONS

This manuscript was conceptualized by all the authors. J.Z. conceived ideas and drafted original draft preparation. F.W. and L.W. edited the manuscript. F.P., C.P., and R.H. administrated funding and revised the manuscript. All authors have read and agreed to the published version of the manuscript.

## CONFLICT OF INTEREST STATEMENT

The authors declare they have no conflicts of interest.

## ETHICS STATEMENT

The tissues microarrays ship of triple‐negative breast cancer samples was collected from National Human Genetic Resources Sharing Service Platform (NHGRP; Shanghai, China; approval ID: 2005DKA21300). Written informed consent was obtained from all participants. The animal experiments were approved by the Ethics Committee of the Animal Research Centre of Chengdu University of Traditional Chinese Medicine (approved ID: 2023004).

## Supporting information

Supporting Information

Supporting Information

Supporting Information

Supporting Information

## Data Availability

The datasets used and/or analyzed during the current study are available from public database or the corresponding author upon reasonable request. All codes are available in GitHub (https://github.com/Jianbo1999).
