# Supplementary figures and images for: STAT4 facilitates PD‐L1 level via IL‐12R/JAK2/STAT3 axis and predicts immunotherapy response in breast cancer
Source: MedComm (2020). 2023 Dec 15;4(6):e464. doi: 10.1002/mco2.464 (PMC10724500; doi:10.1002/mco2.464)

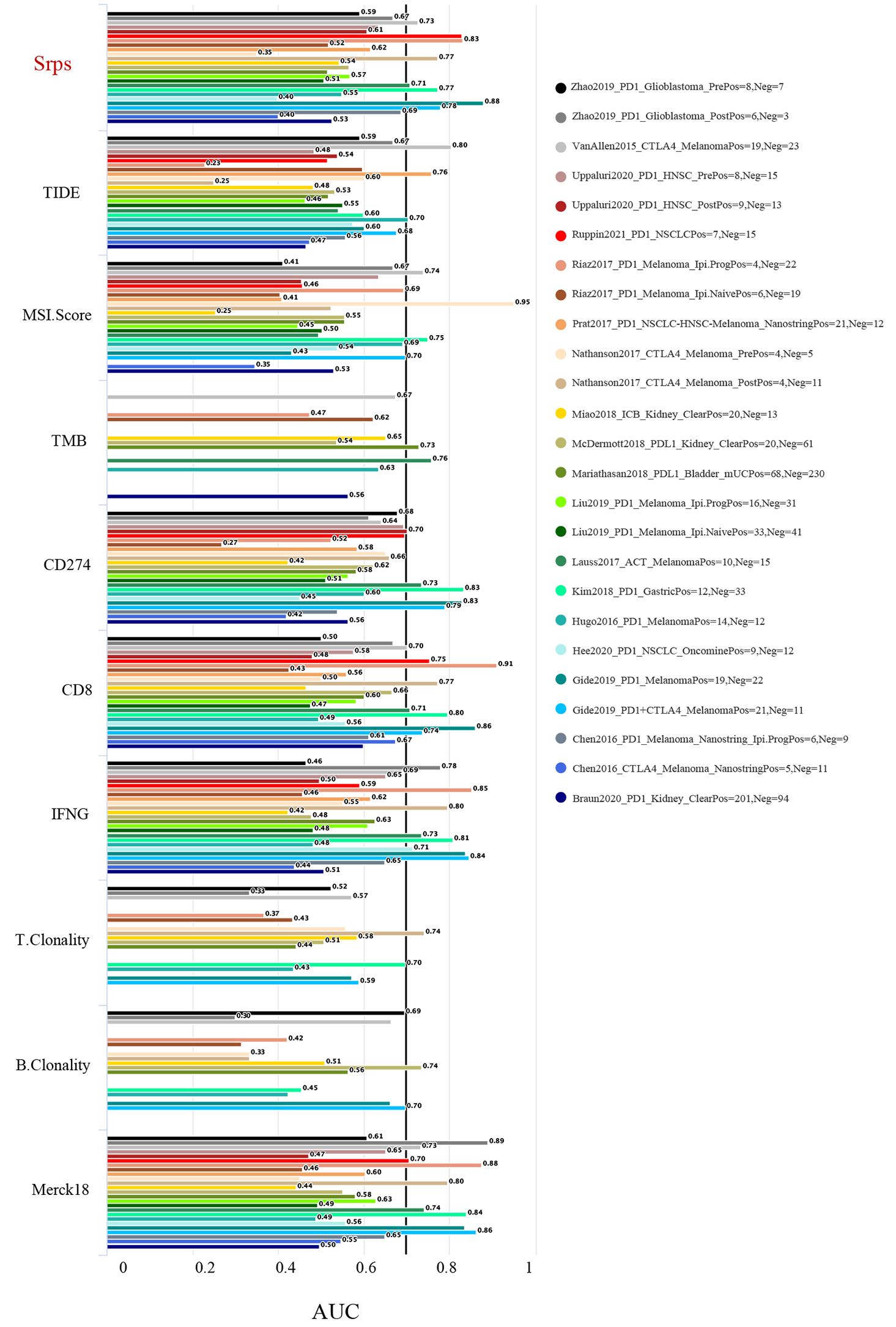

Supplement: Supplementary file 1 — Supporting Information [file MCO2-4-e464-s003.tif]

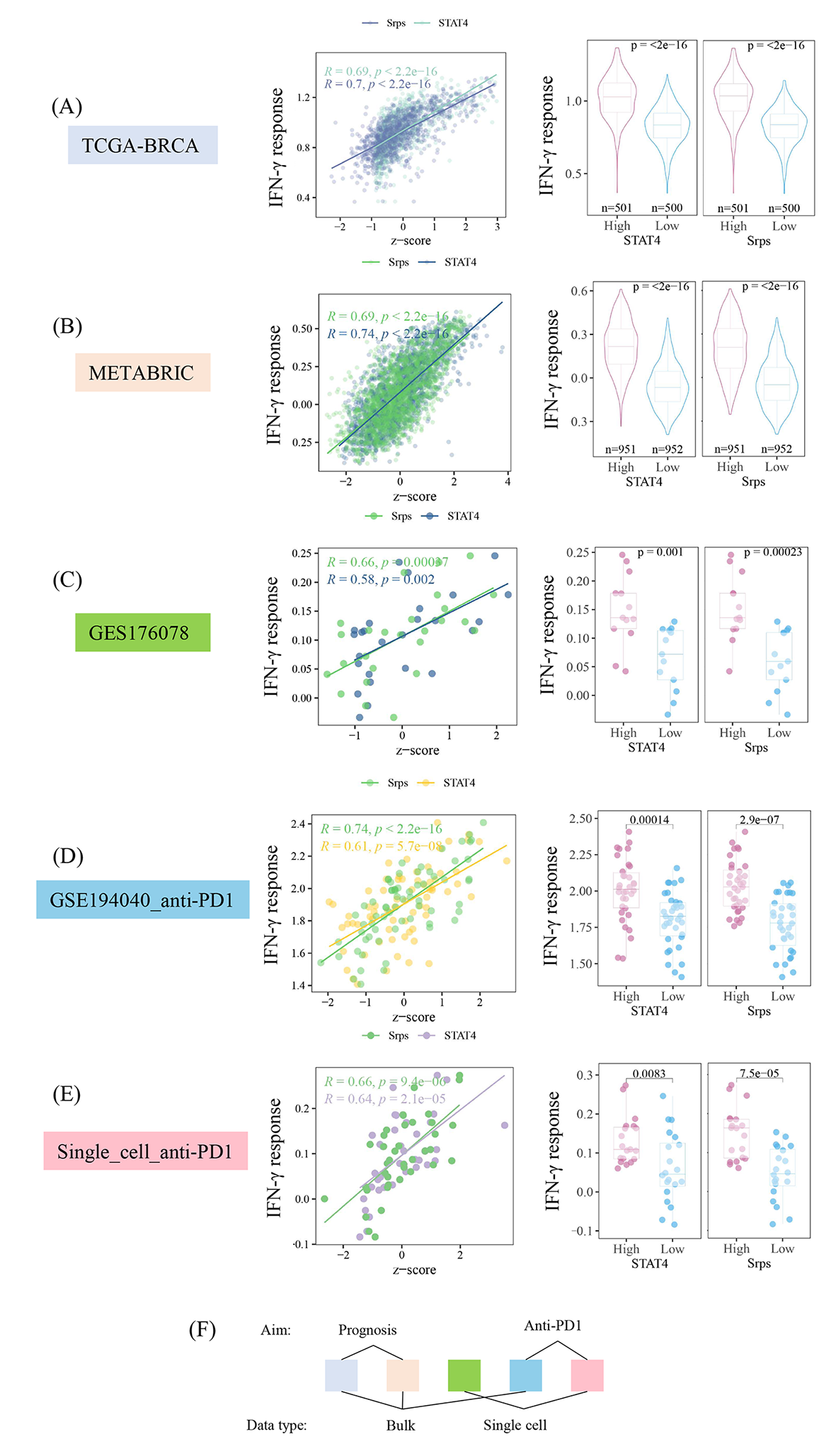

Supplement: Supplementary file 2 — Supporting Information [file MCO2-4-e464-s004.tif]

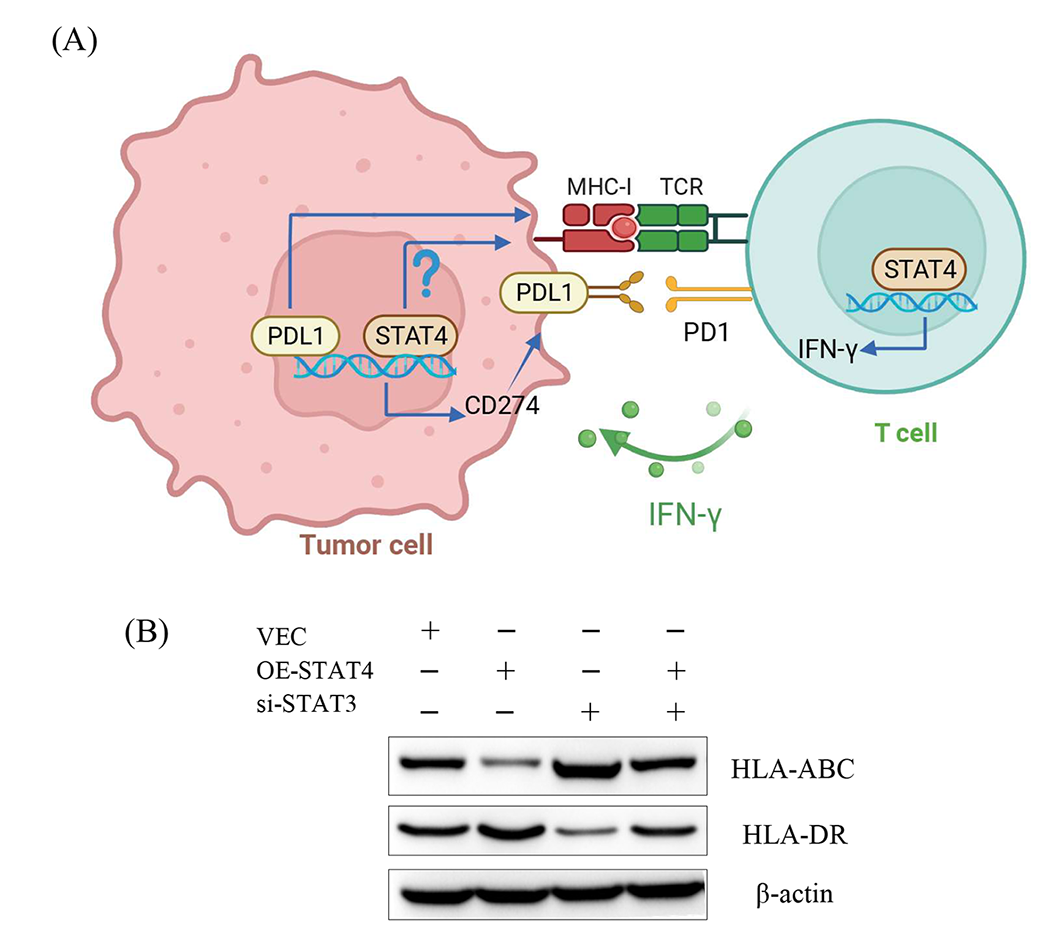

Supplement: Supplementary file 3 — Supporting Information [file MCO2-4-e464-s002.tif]
