# Supplementary material for: STAT4 facilitates PD‐L1 level via IL‐12R/JAK2/STAT3 axis and predicts immunotherapy response in breast cancer
Source: MedComm (2020). 2023 Dec 15;4(6):e464. doi: 10.1002/mco2.464 (PMC10724500; doi:10.1002/mco2.464)
Supplement: Supplementary file 4 — Supporting Information [file MCO2-4-e464-s001.docx]

**Supporting information**

STAT4 facilitates PD-L1 level via IL-12R/JAK2/STAT3 axis and predicts immunotherapy response in breast cancer

Jianbo Zhou ^1^, Feng Wan ^2^，Li Wang ^1^, Cheng Peng ^2,*^, Ruizhen Huang^3,*^_，_ Fu Peng ^1,4,*^

^1^West China School of Pharmacy, Sichuan University, Chengdu, China

^2^State Key Laboratory of Southwestern Chinese Medicine Resources, Chengdu University of Traditional Chinese Medicine, Chengdu, China

^3^Cardiovascular Department, Hospital of Chengdu University of Traditional Chinese Medicine, Chengdu, China

^4^Key Laboratory of Drug-Targeting and Drug Delivery System of the Education Ministry and Sichuan Province, Sichuan Engineering Laboratory for Plant-Sourced Drug and Sichuan Research Center for Drug Precision Industrial Technology, Sichuan University, Chengdu, China

***Correspondence:** Cheng Peng, pengchengchengdu@126.com; Ruizhen Huang, HuangRui-zhen41@foxmail.com; Fu Peng, fujing126@yeah.net.

**Supplementary Table 1** Clinicopathologic characteristics of collected triple negative breast cancer cohort.

|  |  | **Overall** | **STAT4-High** | **STAT4-Low** | ***p* value** |
| --- | --- | --- | --- | --- | --- |
| Characteristics | Cases | 86 | 43 | 43 |  |
| OS status (%) | Alive | 46 (53.5) | 30 (69.8) | 16 (37.2) | **0.005** |
|  | Dead | 40 (46.5) | 13 (30.2) | 27 (62.8) |  |
| age (mean (SD)) | | 53.65 (13.41) | 53.93 (13.21) | 53.37 (13.75) | 0.848 |
| grade (%) | 1 | 77 (89.5) | 39 (90.7) | 38 (88.4) | 1.0 |
|  | 2 | 9 (10.5) | 4 (9.3) | 5 (11.6) |  |
| size (mean (SD)) | | 3.65 (1.97) | 3.47 (1.52) | 3.84 (2.34) | 0.39 |
| T (%) | T1 | 76 (88.4) | 39 (90.7) | 37 (86.0) | 0.737 |
|  | T2 | 10 (11.6) | 4 (9.3) | 6 (14.0) |  |
| N (%) | N0 | 29 (33.7) | 15 (34.9) | 14 (32.6) | 1.0 |
|  | N1 | 57 (66.3) | 28 (65.1) | 29 (67.4) |  |
| Group (%) | High | 43 (50.0) | 43 (100.0) | 0 (0.0) |  |
|  | Low | 43 (50.0) | 0 (0.0) | 43 (100.0) |  |

OS, overall survival; T, tumor stage; N, Node stage; SD, standard deviation. Statistical methods: Fisher's exact test.


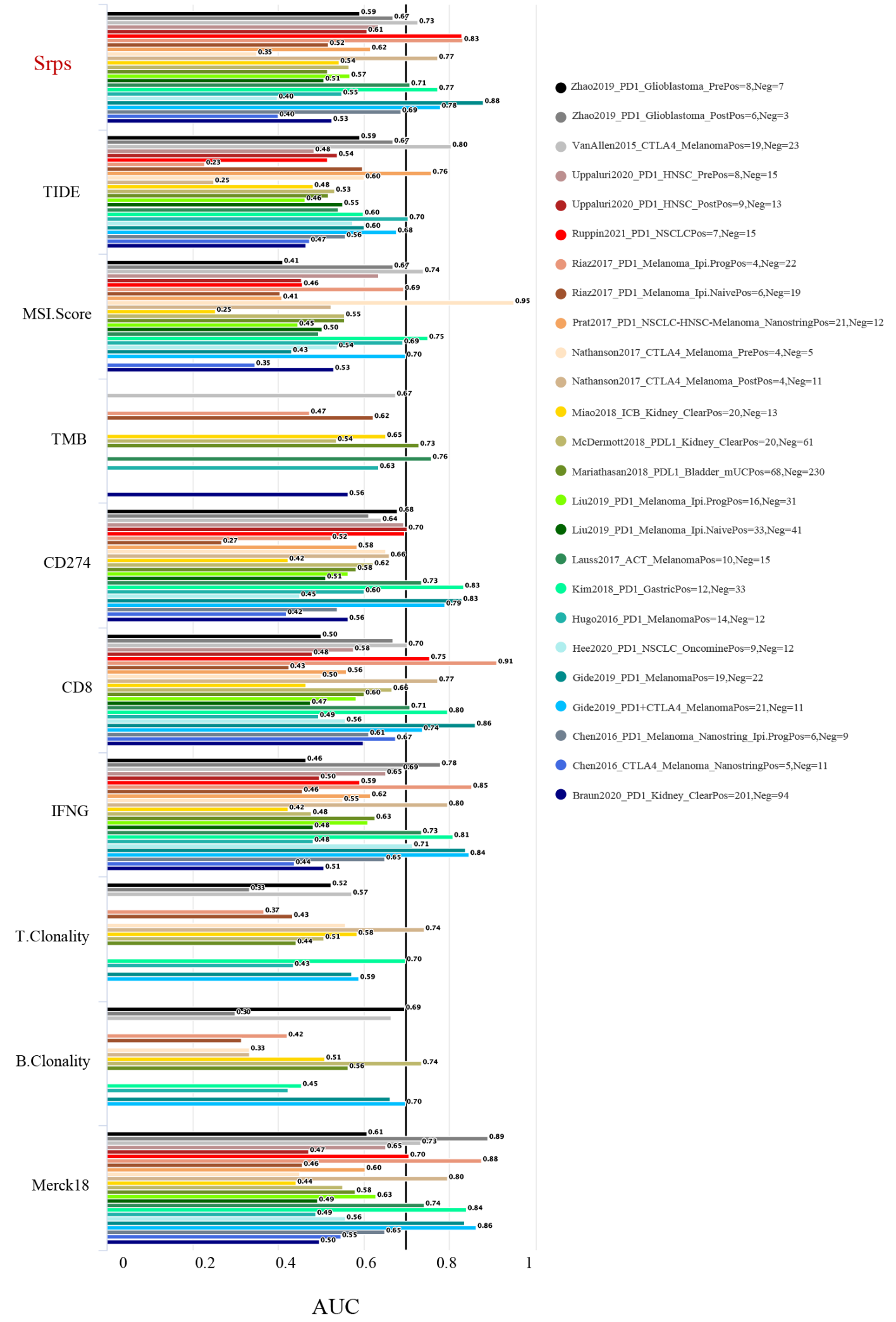


**Supplementary Figure 1** Brocading the predictive effect of Srps on multiple immunotherapy cohorts in TIDE database ^1^. Data are retrieved from TIDE database. The vertical black line means AUC = 0.7.Srps, STAT4 related pathway score; TIDE, tumor immune dysfunction and exclusion score; MSI, microsatellite instability signature; TMB, tumor mutation burden; IFNG, Interferon gamma response biomarkers; CD8, Average expression of CD8A and CD8B; AUC, the area under the receiver operating characteristic curve.


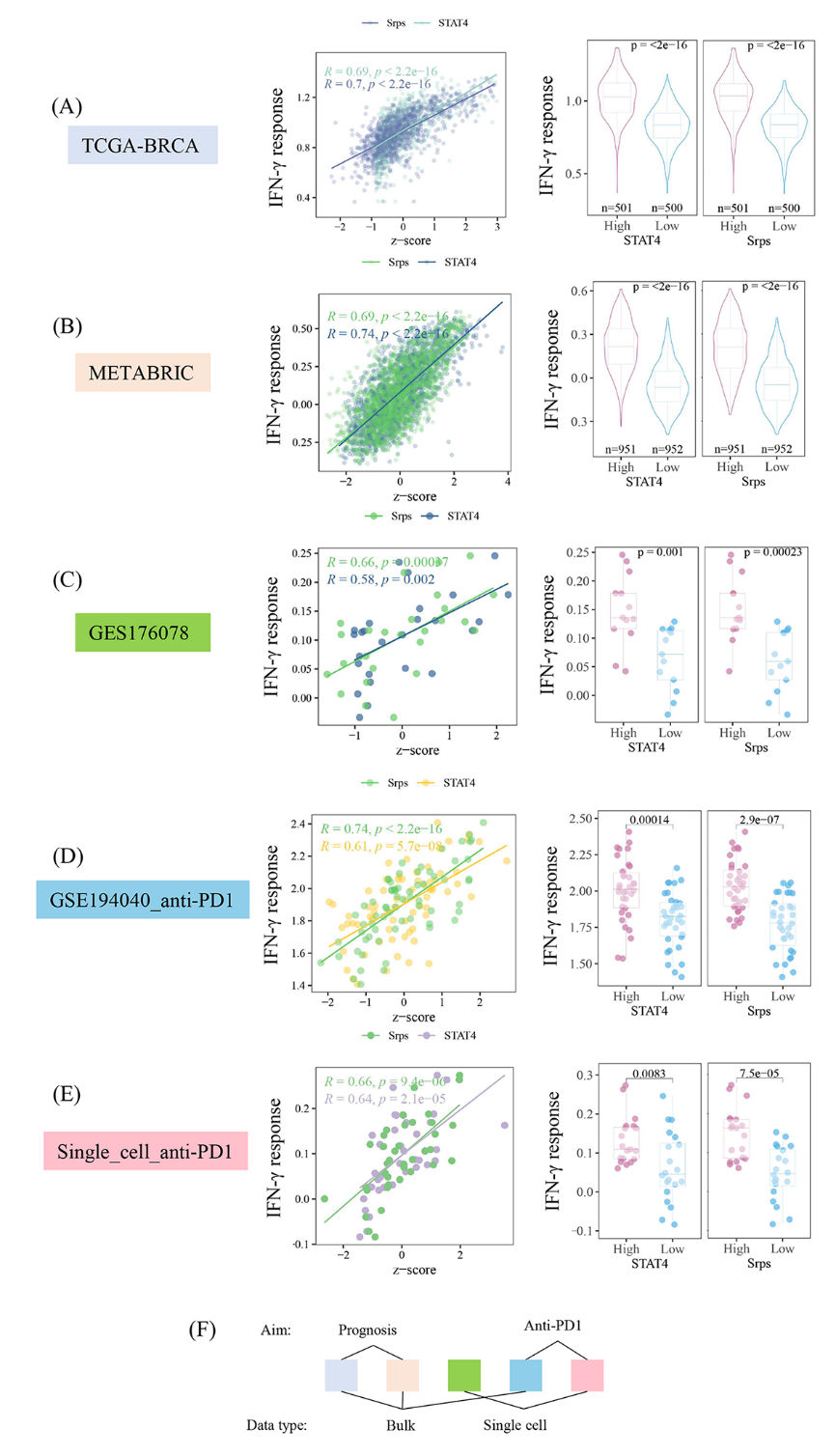


**Supplementary Figure 2** STAT4 is relevant with IFN-γ in breast cancer tumor environment with or without anti-PD1 treatment. IFN-γ response score (HALLMARK_INTERFERON_GAMMA_RESPONSE gene set in MSigDB) was calculated using ssGSEA method and Seurat’s *AddModuleScore* function in bulk sequencing data and in single-cell datasets, respectively ^2^. (A-D) STAT4 and Srps were associated with IFN-γ response score in all datasets of this article. High-STAT4 and high-Srps patients showed higher IFN-γ response.

(F) the using aim and data type of all datasets in context. Single-cell sequencing data also were analyzed for investigating tumor environment.


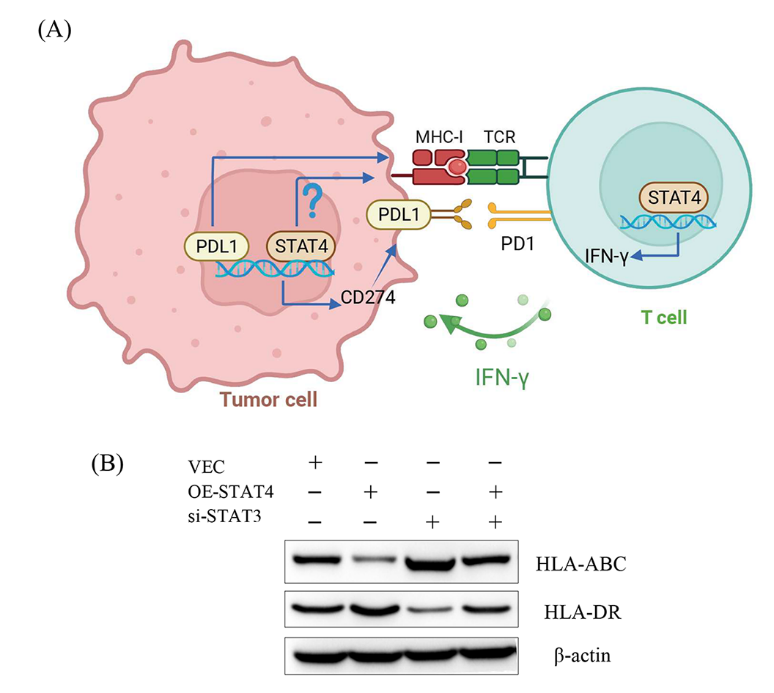


**Supplementary Figure 3** The potential mechanism that STAT4 to benefit prognosis and anti-PD1 therapy involves antigen presentation and IFN-γ response in tumor microenvironment (A). PD-L1 transactivated MHC-I related gene expression (HLA-A, HLA-B, HLA-H) for tumor neoantigen presentation to T cell via MHC-I/TCR axis ^3^. STAT4 augmented IFN-γ release in T cell ^4^, NK cell ^5^ and macrophage ^6^. Moreover, IFN-γ has been recognized for its anti-tumor activity and immunosurveillance ^7^. However, it is unclear whether STAT4 directly transactivates MHC-I in breast cancer. MHC class I and class II molecules present peptides at the cell surface to CD8+ and CD4+ T cells, respectively ^8^.Tumor cells escape T-cell surveillance via downregulating MHC proteins to reduce antigen presentation ^9^. Herein, STAT4 upregulated MHC-II (HLA-DR) level, while si-STAT3 increased MHC-I (HLA-ABC) expression in cancer cells (B). These results suggest STAT4 over-expression and / or silencing STAT3 may serve as a strategy for MHC-induced T-cell response.

**References:**

1. Jiang, P.; Gu, S.; Pan, D.; Fu, J.; Sahu, A.; Hu, X.; Li, Z.; Traugh, N.; Bu, X.; Li, B.; et al. Signatures of T cell dysfunction and exclusion predict cancer immunotherapy response. *Nature Medicine* **2018**, *24*, 1550-1558, doi:10.1038/s41591-018-0136-1.

2. Bassez, A.; Vos, H.; Van Dyck, L.; Floris, G.; Arijs, I.; Desmedt, C.; Boeckx, B.; Vanden Bempt, M.; Nevelsteen, I.; Lambein, K.; et al. A single-cell map of intratumoral changes during anti-PD1 treatment of patients with breast cancer. *Nature Medicine* **2021**, *27*, 820-832, doi:10.1038/s41591-021-01323-8.

3. Gao, Y.; Nihira, N.T.; Bu, X.; Chu, C.; Zhang, J.; Kolodziejczyk, A.; Fan, Y.; Chan, N.T.; Ma, L.; Liu, J.; et al. Acetylation-dependent regulation of PD-L1 nuclear translocation dictates the efficacy of anti-PD-1 immunotherapy. *Nature Cell Biology* **2020**, *22*, 1064-1075, doi:10.1038/s41556-020-0562-4.

4. Anderson, K.; Ryan, N.; Volpedo, G.; Varikuti, S.; Satoskar, A.R.; Oghumu, S. Immune Suppression Mediated by STAT4 Deficiency Promotes Lymphatic Metastasis in HNSCC. *Frontiers in Immunology* **2020**, *10*, doi:10.3389/fimmu.2019.03095.

5. Gotthardt, D.; Trifinopoulos, J.; Sexl, V.; Putz, E.M. JAK/STAT Cytokine Signaling at the Crossroad of NK Cell Development and Maturation. *Frontiers in Immunology* **2019**, *10*, doi:10.3389/fimmu.2019.02590.

6. Xiao, J.; Sun, F.; Wang, Y.-N.; Liu, B.; Zhou, P.; Wang, F.-X.; Zhou, H.-F.; Ge, Y.; Yue, T.-T.; Luo, J.-H.; et al. UBC9 deficiency enhances immunostimulatory macrophage activation and subsequent antitumor T cell response in prostate cancer. *The Journal of Clinical Investigation* **2023**, *133*, doi:10.1172/JCI158352.

7. Burke, J.D.; Young, H.A. IFN-γ: A cytokine at the right time, is in the right place. *Seminars in Immunology* **2019**, *43*, 101280, doi:10.1016/j.smim.2019.05.002.

8. Neefjes, J.; Jongsma, M.L.; Paul, P.; Bakke, O. Towards a systems understanding of MHC class I and MHC class II antigen presentation. *Nat Rev Immunol* **2011**, *11*, 823-836, doi:10.1038/nri3084.

9. Schaafsma, E.; Fugle, C.M.; Wang, X.; Cheng, C. Pan-cancer association of HLA gene expression with cancer prognosis and immunotherapy efficacy. *British journal of cancer* **2021**, *125*, 422-432, doi:10.1038/s41416-021-01400-2.
